# Diagnosing Arthrogryposis Multiplex Congenita: A Review

**DOI:** 10.5402/2012/264918

**Published:** 2012-09-23

**Authors:** Emmanouil Kalampokas, Theodoros Kalampokas, Chrisostomos Sofoudis, Efthymios Deligeoroglou, Dimitrios Botsis

**Affiliations:** Second Department of Obstetrics and Gynecology, Aretaieion University Hospital, University of Athens, 76 Vas. Sofias Avenue, 11528 Athens, Greece

## Abstract

Arthrogryposis multiplex congenita (AMC) refers either to a syndromic or to a nonsyndromic group of conditions with varied etiology and complex clinical features, including multiple congenital contractures in different body areas. Its etiology still remains unclear but generally any cause that leads to reduced fetal movement may lead to congenital contractures and in severe cases to fetal akinesia deformation sequence (FADS). It affects approximately 1 in 2-3000 live births with an approximately equal gender ratio. There are many known subgroups of AMC differing in signs, symptoms, and causes. The primary diagnosis is made when a lack of mobility and an abnormal position is noted in routine ultrasound scanning. Early diagnosis, prenatal evaluation, and further surveillance via image scanning (ultrasound and MRI) give the opportunity for family counseling concerning neonatal morbidity and mortality and labor or delivery planning. Better understanding of the ultrasound findings and the etiology of this clinical situation offers the opportunity for careful prenatal assessment.

## 1. Introduction

Congenital contracture is a muscle condition present from birth and refers to an abnormal and usually permanent contraction of muscle fibers, with a concomitant inability of passive extension and flexion and may be due to prolonged immobilization, serious injuries, and musculoskeletal or circulatory disorders containing isolated contractures and multiple contractures. Approximately 1% of all live births show some type of contractures in one or more joints ranging in severity from unilateral clubfoot to fetal akinesia or the classic Pena-Shokeir phenotype (perinatal lethal form of multiple contracture conditions) [1].

Arthrogryposis multiplex congenita (AMC) consists of several conditions of different etiology and mixed clinical features, including multiple congenital contractures in multiple body areas. The etiology still remains unclear but generally any cause that leads to reduced fetal movement may guide to congenital contractures and in severe cases to fetal akinesia deformation sequence (FADS) because proper fetal growth is dependent on fetal movement, starting by 8 weeks' gestation [2, 3]. For practical reasons, it can be divided into subgroups, as a way of generating a differential diagnosis which includes neurological diseases (brain, spine, or peripheral nerve), connective tissue defects (diastrophic dysplasia), muscle abnormalities (muscular dystrophies or mitochondrial abnormalities), space limitations within the uterus (oligohydramnios, fibroids, uterine malformations, or multiple pregnancy), intrauterine or fetal vascular compromise (impaired normal development of nerves, or anterior horn cell death), and maternal diseases (diabetes mellitus, multiple sclerosis, myasthenia gravis, infection, drugs, or trauma) [4–6]. It should be also noted that arthrogryposis could be a clinical manifestation of different syndromes such as dysgenesis of the nervous system observed in chromosome abnormalities (trisomy 18 and 21) and dysplasias of brainstem nuclei and spinal cord as seen in the Möbius, Pierre-Robin, prune belly, and Zellweger syndrome [7]. It affects approximately 1 in 2-3000 live births [2, 8] or 1 in 5–10000 live births according to other authors [9, 10] with an approximately equal gender ratio.

What is more, connective tissue deposition is observed around joints due to intrauterine fetal movement restriction, resulting in fixation and contractures or skeletal abnormalities [2]. The above-mentioned joint abnormalities may involve all limbs (usually) in the lower or the upper extremities. Arthrogryposis is usually symmetrical, but, less frequently, various joints may be involved to a different extent [11]. 

## 2. Arthrogryposis Multiplex Congenital (AMC)

AMC is a rare sporadic nonprogressive congenital disorder that is characterized by multiple joint contractures and can incorporate muscle weakness and fibrosis. The disease name derives from Greek, meaning “curved or hooked joints.” Research has shown that anything that inhibits normal joint movement before birth can result in joint contractures since tendons connecting to the joint are not stretched to their normal length.

### 2.1. Etiology

In animal models, viruses, neuromuscular diseases, hyperthermia, and limb immobilization are responsible for contractures [1]. In spite of the fact that fetal akinesia in humans is the major cause of AMC, there are multiple and varied intrinsic as well as extrinsic causes for reduced fetal movements. Categorization in groups of causes that are responsible for impaired fetal movement are described by Hall [4]. 

#### 2.1.1. Neurologic Abnormalities

Neurologic abnormalities seem to be one of the most common causes of AMC (approximately 70–80% of cases). The reason for this is brain disorders shown by magnetic resonance imaging (MRI), prenatal ultrasound (in order to define intracranial pathology), and later at postpartum autopsies like epilepsy, defects in neural migration, cerebral hypoplasia, holoprosencephaly, pyramidal tract degeneration, and olivoponto-cerebellar degeneration [8, 12–18] which can be associated with chromosomal aneuploidy, an underlying genetic syndrome or the result of a teratogen [1]. Anterior horn cell disease (including the Werdnig-Hoffmann disease) is one of the most common causes of spinal cord degeneration (autosomal inheritance) [13] with spinal muscular atrophy being a close second neurogenic cause of arthrogryposis [14, 19]. Last but not least, peripheral neuropathy has been closely associated with AMC [20] because of medium-to-large-axon demyelination as a result of deficiency of the myelin proteins P2 and P0, myelin basic protein, and myelin-associated glycoprotein showing that arrest of peripheral myelination at the promyelin stage appears to be the origin of myelin deficiency [21]. In addition, failure in spinal lengthening and longitudinal growth of Schwann cells could also lead to AMC [22].

#### 2.1.2. Muscle Abnormalities

To begin with, an association has been strongly linked between AMC and myasthenia mainly because of maternal antibodies entering fetal circulation through transplacental transfer and thus inhibiting fetal acetylcholine receptor function leading to damage to fetal muscle, impaired fetal movement *in utero*, and the development of multiple joint contractures [23–25]. For example, a large number of neonatal cases in women with myasthenia gravis has been reported with hypotonia, extraocular weakness, bulbar symptoms, respiratory distress, and multiple joint contractures [26–28].

Furthermore, disorders arriving from the muscles like muscular dystrophy, myopathies, myositis, and mitochondrial disorders are common causes of AMC. These include central core disease, nemaline myopathy, intranuclear rod myopathy, and many other types of congenital myopathies [1, 29, 30] caused by a mutation in the gene encoding for sarcomeric thin filament protein troponin I [31] or a deficiency of *α*-actinin-3 [32]. Congenital muscular dystrophies (1–10000 live births) are the result of abnormal function of the dystrophin-glycoprotein-associated complex in the sarcolemma of skeletal muscles [1]. In addition, mitochondrial cytopathy is also considered to play a major role in AMC [33]. The pathophysiologic mechanism of this is located in the area of muscle fibers (ragged-red fibers), central nervous system, and chondrocytes [34, 35].

#### 2.1.3. Connective Tissue Abnormalities

AMC may also result from connective tissue abnormalities because of a collagenic response (law of connective tissue) on account of the resultant loss of muscle mass with an imbalance of muscle power at the joints. The collagenic response consists of partial replacement of muscle volume and collagenous thickening of the joint capsules leading to joint fixation [36, 37], subsequent reduced fetal movement, and contractures observed in pterygium syndrome [4, 13, 38], congenital contractural arachnodactyly, Beals syndrome (congenital heart disease, scoliosis, arachnodactyly, and “crumpled ears”), and Larsen syndrome (anterior dislocation of the knees) [1, 39].

#### 2.1.4. Intrauterine Space Constraint, Vascular Compromise, Maternal Disease, and Teratogenic Exposure

Amniotic cavity filled with amniotic fluid protects the fetus from extrinsic hazardous factors and provides the adequate space for proper fetal development and movement. Pathologies arriving from the volume of amniotic fluid like oligohydramnios (which commonly results from leakage of amniotic fluid due to cervical incompetence or due to Potter's syndrome (bilateral renal agenesis)) are some of the major causes of space limitation hence leading to contractures which are more severe the earlier they happen [1, 13, 40, 41]. Furthermore, amniocentesis before 15 weeks of gestation has been reported to have a 10-fold higher risk for multiple contractures and clubfoot [1]. Moreover, this can also be observed in congenital uterine deformities, fibroids, uterine tumors, and multiple pregnancy [4] (as it is known that incidence of arthrogryposis is more common among twins than singletons [7]). 

Next, inadequate vascular supply to the fetus causes fetal hypoxia that leads to anoxic injury of tissue and/or blood clots or blockage of blood flow resulting in possible cell death, particularly anterior horn cell death or failure. Inferior anterior horn cell function would likely cause fetal neuron, muscle, and bone damage and secondary multiple joint contractures [7, 41]. In addition, maternal disease, such as diabetes mellitus, multiple sclerosis, myotonic dystrophy, and infections (such as rubella, varicella, equine encephalitis, cytomegalovirus, and toxoplasmosis) have been closely related with fetal akinesia and subsequent AMC. However, in many cases this cannot be proved if it was causal rather than coincidental [4, 7, 42–45]. Lastly, medical administration or drug abuse during pregnancy, for instance of curare (a skeletal muscle relaxant), misoprostol, or substances such as cocaine and alcohol, can result in congenital contractures if given at a critical period of fetal development [46, 47]. 

### 2.2. Classification

There are many known subgroups of AMC differing in signs, symptoms, and causes. The principal cause of AMC is both genetic and environmental factors, occurring individually or with a significant overlap among them. In order to establish a differential diagnosis in early child life, it is crucial to determine first if a child has normal neurological function or not. A normal one suggests that arthrogryposis is a result of amyoplasia (the most common recognizable form of AMC which is a sporadic symmetric syndrome that is characterized by the symmetrical improper development of limb muscles which are replaced by fatty and connective tissue and often a midline hemangioma), distal arthrogryposis (an autosomal-dominant inherited syndrome with a characteristic involvement of distal joints with sparing of the large joints; the upper limbs show ulnar deviation, camptodactyly, hypoplastic, or absent flexion and overriding fingers whereas the lower ones can show talipes equinovarus, calcaneovalgus, vertical talus, and metatarsus varus), a systemic connective tissue disorder, multiple pterygium syndromes (a group of autosomal dominant, recessive, or X-linked inherited syndromes manifested with multiple contractures (“pterygia”), micrognathia, low-set ears, cardiac and lung hypoplasia, cystic hygroma, and hydrops), or fetal crowding [1, 11, 39]. On the contrary, an abnormal neurological function highlights that diminished fetal movement *in utero* was the result of an abnormality of the central or peripheral nervous system, the motor end plate, or the muscles [48] (Table 1).

### 2.3. Ultrasound Scanning Findings

Although fetal movement is observed by ultrasound scan at 8 weeks' gestation, most cases of AMC are diagnosed prenatally at the second or third trimester of pregnancy with ultrasound scan and/or with the combination of maternal consideration for reduced fetal *in utero* movements. The combination of maternal consideration for fetal akinesia with ultrasound abnormalities (usually before the perception of fetal movement) will give the prospect of an arthrogrypotic syndrome [2, 42, 49]. The earlier the contractures occur (or if there is a syndrome present with abnormalities other than skeletal malformations), the harder early prenatal diagnosis will be [42]. 

The primary diagnosis is made when a lack of mobility and an abnormal position is noted in routine ultrasound scanning. These findings should guide the practitioner to a careful assessment of fetal anatomy and joints. The most common detailed ultrasound scan findings are fixed flexion deformities, micrognathia, altered amniotic fluid volume, limb deformities, cerebral ventriculomegaly, dysmorphic features, and growth retardation [2]. AMC may also be present with increased nuchal translucency at 10–14 weeks [49] or increased nuchal translucency and scoliosis at 15 weeks [50] of pregnancy due to nuchal edema that has been found in first and early second trimester cases of arthrogryposis [51, 52], cystic hygroma [42] and fetal seizures [53] on first trimester ultrasound, small chest, thin ribs, and multiple diaphyseal fractures [54]. Visualization of details of the dynamics of small anatomical structures can now be done better and earlier (body and limb movements can be visualized a week earlier than with 2D) with the use of 4D ultrasound giving the possibility of a diagnosis of motoric failure by the end of the first trimester [55] (Table 2).

### 2.4. Signs and Symptoms

Some of the more common signs and symptoms are associated with the shoulder (internal rotation), elbow (extension and pronation), wrist (volar and ulnar), hand (fingers in fixed flexion and thumb in palm), hip (flexed, abducted and externally rotated, often dislocated), knee (flexion), and foot (clubfoot). In some cases, a small number of joints may be affected and may have an almost full range of motion capabillity but in the most severe types, nearly every joint is involved, including the jaw and back [11, 56]. Furthermore, complications may include various congenital anomalies from the organs that can be related with arthrogryposis such as scoliosis, lung hypoplasia, respiratory problems, growth retardation, midfacial hemangioma, facial and jaw variations and abdominal hernias, congenital heart defects, tracheoesophageal fistulas, and ophthalmologic abnormalities [57] (Table 3).

## 3. Discussion

Multiple contractures in general and AMC more specifically are a common ultrasound finding although they are described as clinical findings rather than a precise diagnosis. Neurogenic, or myopathy disorder, connective tissue process, intrauterine compression, vascular compromise, or teratogenic exposure could be the principal cause of the ultrasound finding [1].

Early diagnosis, prenatal evaluation, and further surveillance via image scanning (ultrasound and MRI) give the opportunity for family counseling concerning elevated neonatal morbidity and mortality in these cases and labor or delivery planning. Patients should be also informed that a specific prenatal diagnosis of arthrogryposis reaches only up to 50% [3]. Craniofacial abnormalities and micrognathia more often seems to be the consequence of neuromuscular dysfunction because of the adequate growth for the craniofacial bones, fetal muscle movement is required [45, 51]. 

Diagnosis of the condition by ultrasound will depend on the gestational age and possible presence of other anomalies, with additional MRI surveillance providing additional information such as distal muscle atrophy, abnormal muscle formation, and lung volume measuring [58, 59]. In several previous studies, the focus has been given to cystic hygroma and increased nuchal translucency with combination of reduced fetal movements *in utero* leading in this way to an early diagnosis of arthrogryposis in first and/or early second trimester of pregnancy [42, 49, 50, 60].

Other studies found that in some cases arthrogryposis occurring with hydramnion and rarely with hydrops may be a result of impaired swallowing [46, 49, 60, 61].

The importance of autopsy and tissue procurement should be noted to parents as it can confirm prenatal diagnosis and provide families with valuable information other than that given by ultrasound and MRI scanning [62]. 

## 4. Conclusion

Congenital contractures are a common birth defect and a subsequently prenatal ultrasound finding, observed more often randomly in early second trimester ultrasound prenatal assessment. Any factor that predisposes the fetus to impaired fetal movement can cause congenital contractures, such as environmental, maternal, and genetic factors. AMC is a term that describes contractures in multiple joints, in more than one area of the body, associated with fetal morbidity and future economic burden to put right. Better understanding of ultrasound findings and the etiology of this clinical situation offers an opportunity for careful prenatal assessment through thorough image scanning focusing on flexion/extension, position of proximal and distal joints, jaw, and spine. When prenatal diagnosis is suspected families should be counseled for potential postnatal or postterm evaluation.

## Figures and Tables

**Table 1:** Arthrogryposis classification.

Normal neurological function	Amyoplasia	Distal arthrogryposis	Fetal crowding	Systemic tissue disorder	Multiple pterygium disorders
Abnormal neurological function					

**Table 2:** Ultrasound findings.

Lack of mobility	
Abnormal position	
Fixed flexion deformities	
Cerebral ventriculomegaly	
Dysmorphic features	
Growth retardation	
Increased nuchal translucency	
Scoliosis	
Cystic hygroma	
Fetal seizures	
Small chest	
Thin ribs	
Multiple diaphyseal fractures	

**Table 3:** Signs and symptoms.

Shoulder	Internal rotation
Elbow	Extension-pronation
Wrist	Volar-ulnar
Hand	Fingers-fixed flexion, thumb in palm
Hip	Flexed, abducted, and externally rotated
Knee	Flexion
Foot	Clubfoot
Joints	
Various	Scoliosis, Lung hypoplasia, respiratory problems, growth retardation, midfacial hemangioma, facial-jaw variations, abdominal hernias, congenital heart defects, tracheoesophageal fistulas, ophthalmologic abnormalities
